# Supplementary material for: Patterns of Intron Gain and Loss in Fungi
Source: PLoS Biol. 2004 Nov 30;2(12):e422. doi: 10.1371/journal.pbio.0020422 (PMC532390; doi:10.1371/journal.pbio.0020422)
Supplement: Table S1 — Also available at http://genes.mit.edu/NielsenEtAl/. (4.3 MB ZIP). [file pbio.0020422.st001.zip › NielsenEtAl/html/869.html]

AN5529.1.NCU07296.1.MG05376.1.FG01188.1


```
 CLUSTAL W (1.82) Multiple Sequence Alignments - Introns Inserted


Sequence 1: NCU07296.1	613 aa
Sequence 2: MG05376.1	416 aa
Sequence 3: FG01188.1	609 aa
Sequence 4: AN5529.1	588 aa
Alignment Length: 655 aa
Number Identitical Residues: 262 aa
Alignment Score (without introns) 13182


MG05376.1 	------------------------------------------------------------
NCU07296.1	MD---NTNRPHLNLGTNDTRMAPNDRT-YPTTPSTFPQPVFP----GQQAGGSQ------
FG01188.1 	MDPNNSNNRLYLNFGNGNDRLTASDRTAYPTTPSTFPQPVFPSASSGQQSGGLQPQQQQQ
AN5529.1  	--------------------MDAQNG----------------------------------
          	                      ...                                   

MG05376.1 	------------------------------------------------------------
NCU07296.1	-QYNQAYAQSGNYYQQNQYASHYAP-PGAADYGF---VQAHSNIDGTSNDPNTGLAHQFA
FG01188.1 	QQFAGGYAPQG-YFNQNQYAQYGGQQPQQNDYQQGAGYQQRSNTPG-TNDPNTGLAHQFS
AN5529.1  	-VYGQGYFMPNNYPAQAAYAQPHYGQPNLQSPQP--AYQSRMGYNVSPNDGTNGLIQQFS
          	     .    ..   .   :.    ..   .     . .   .   :... ...   . :

MG05376.1 	------------------------------------~-----------------------
NCU07296.1	HQNIGSAGRASPYGSRGPSPAQRPRTSGNSGQ-QQT~YGNYLSAPMPSNTQTEFA-PAPE
FG01188.1 	HQNLGGAARGQAY-ARGPSPGQRPRTAGSSGQ-QPD1-----MAPTPTATR---------
AN5529.1  	NQDLNSNR--TGFFNRSASPAQRPRTAGNTAPGQQQ~QPGHLAPPVPRSPRLPPENEELQ
          	 .. ..          ..:... . ::..:. ..     .   .. .  .     .   .

MG05376.1 	--------------------------------------~---MELKLSEPNQSQSRKEQI
NCU07296.1	RNPDKYGPNANNNQKKCSQLASDFFKDSVKRARERNQR2QSEMEQKLGETNDAR-RRESI
FG01188.1 	-------RNALSLLPTSSRIVSREHASATK-------E1QSELEQKLQDPTQGAARREQL
AN5529.1  	RYPERFSENVHKRGKAAKELVNVFFHENIERARDRNMR2SAELDKMMRDPNISQDAKVKE
          	  ..  . .  .    ..   .    .  . : . .   .:.::  : :.. .   : . 

MG05376.1 	WSTGGRKEGSYLRFLRTKDKPENYNTIKIIGKGAFGEVKLVQKKSDNKVYAMKSLIKTEM
NCU07296.1	WSTAGRKEGQYLRFLRTKDKPENYQTIKIIGKGAFGEVKLVQKKADGKVYAMKSLIKTEM
FG01188.1 	WSTAGRKEGQYLRFLRTKDKPENYNTVKIIGKGAFGEVKLVQKKGDGKVYAMKSLIKTEM
AN5529.1  	AEMVGKKESTFLRFLRTPETPANFQTIKIIGKGAFGEVKLVQRKSDNKIYALKSLIKSEM
          	 .  *:**. :****** :.* *::*:***************:*.*.*:**:*****:**

MG05376.1 	FKKDQLAHVRAERDILAES-DSPWVVKLFTTFQDANFLYMLMEFLPGGDLMTMLIKYEIF
NCU07296.1	FKKDQLAHVRAERDILAES-DSPWVVKLYTTFQDANFLYMLMEFLPGGDLMTMLIKYEIF
FG01188.1 	FKKDQLAHVRSERDILAES-DSPWVVKLYTTFQDSYFLYMLMEFLPGGDLMTMLIKYEIF
AN5529.1  	FKKDQLAHVRAERDILADSKDNPWLVKLHASFQDPAYLYLLMEFLPGGDLMTMLIKYEIF
          	**********:******:*.*.**:***.::***. :**:********************

MG05376.1 	SEDITRFYIAEIVLAIEAVHKLGFIHR2-----------GGHVKLTDFGLSTGFHKLHDN
NCU07296.1	SEDITRFYIAEIVLAIDAVHKLGFIHR2-----------GGHVKLTDFGLSTGFHKLHDN
FG01188.1 	SEDITRFYIAEIVLAIEAVHKLGFIHR2DIKPDNILLDRGGHVKLTDFGLSTGFHRLHDN
AN5529.1  	SEDITRFYMAEIVMAIEAVHKLGFLHR2DIKPDNILLDRGGHVKLTDFGLSTGGKKTHDN
          	********:****:**:*******:** . ....   . ************** :: ***

MG05376.1 	NYYQQLLQGKSNRPRGDRNSIAIDQINLTVSNRAQINDWRRSRRLMAYSTVGTPDYIAPE
NCU07296.1	NYYTQLLQGKSNKPRDNRNSVAIDQINLTVSNRAQINDWRRSRRLMAYSTVGTPDYIAPE
FG01188.1 	NYYQQLLQGRSNKPRD-RNSVAIDQINLTVSNRSQINDWRRSRRLMAYSTVGTPDYIAPE
AN5529.1  	SYYQNLLKNSTSKDKNRNSGYFNDAINLTVSNRGQINTWRKSRRAMAYSTVGTPDYIAPE
          	.** :**:. :.: :. ...   * ********.*** **:*** ***************

MG05376.1 	IFTGHGYTFDCDWWSLGTIMFECLVGWPPFCAEDSHDTYRKIVNWRQSLYFPDDIQLGVE
NCU07296.1	IFTGHGYSFDCDWWSLGTIMFECLVGWPPFCAEDSHDTYRKIVNWRHSLYFPDDITLGVD
FG01188.1 	IFTGHGYTFDCDWWSLGTIMFECLVGWPPFCAEDSHDTYRKIVNWRQTLYFPDDITLGTD
AN5529.1  	IFNGQGYTYLCDWWSVGAIMFECLVGWPPFCAEDTTDTYRKIVNWRECLYFPEELTLSRE
          	**.*:**:: *****:*:****************: **********. ****::: *. :

MG05376.1 	AENLIRS2LICNTENRLGR-----GGAHEIKSHSFFRGVEFEGLRRIRAPFEPRLTSAID
NCU07296.1	AENLIRS2LICNTENRLGR-----GGAHEIKSHAFFRGVEFDSLRRIRAPFEPRLTSAID
FG01188.1 	AEHLIRS2MVCNTENRLGR-----SGAHEIKSHAFFRGVEFDSLRRIRAPFEPRLTSNID
AN5529.1  	SEGLIRS2FLCDAEHRIGNEGGQYGGATQIKNHPFFRGVVWDQLRKIRAPFEPRLTSNID
          	:* **** ::*::*:*:*..... .** :**.*.***** :: **:*********** **

MG05376.1 	TTYFPTDEIDQTDNATVLRAQQMQQQATTGAPVREETVEMSLPFIGYTFKRFDNNFR--
NCU07296.1	TTYFPTDEIDQTDNATLLKAQQAARGAAAPAQQ-EESPELSLPFIGYTFKRFDNNFR--
FG01188.1 	TTYFPTDEIDQTDNATVLKAQAIQSG-----RQVEESPEMSLPFIGYTFKRFDNNFR--
AN5529.1  	VSYFPIDEIPQEDTSAIHRAQARAMPD-------EQNAEMSLPFIGYTYKAFN-AFQAS
          	.:*** *** * *.::: :**             *:. *:********:* *:  *:::
```
